# Epithelial differentiation mimicking tumor-to-tumor metastasis in an isocitrate dehydrogenase wild-type glioblastoma

**DOI:** 10.1093/noajnl/vdae081

**Published:** 2024-05-17

**Authors:** Tomasz Gruchala, Heather Smith, Osaama Khan, Lawrence Jennings, Lucas Santana-Santos, Erica Vormittag-Nocito, Craig Horbinski

**Affiliations:** Department of Pathology, Feinberg School of Medicine, Northwestern University, Chicago, Illinois, USA; Department of Pathology, Feinberg School of Medicine, Northwestern University, Chicago, Illinois, USA; Department of Neurosurgery, Feinberg School of Medicine, Northwestern University, Chicago, Illinois, USA; Department of Pathology, Feinberg School of Medicine, Northwestern University, Chicago, Illinois, USA; Department of Pathology, Feinberg School of Medicine, Northwestern University, Chicago, Illinois, USA; Department of Pathology, Feinberg School of Medicine, Northwestern University, Chicago, Illinois, USA; Department of Neurosurgery, Feinberg School of Medicine, Northwestern University, Chicago, Illinois, USA; Department of Pathology, Feinberg School of Medicine, Northwestern University, Chicago, Illinois, USA

Glioblastoma, isocitrate dehydrogenase wild type (IDH^wt^) is the most common malignant primary brain tumor and exhibits significant heterogeneity between and within tumors.^1^ This can include differentiation into alternative cell lineages, such as epithelial/carcinoma morphology and immunohistochemistry.^2^ In this case report, we describe a patient with glioblastoma, IDH^wr^ containing islands of epithelial differentiation that on first appearance resembled tumor-to-tumor metastasis, another phenomenon that can occur in gliomas.^3^ Molecular testing (including methylation profiling) of both glioma-appearing and carcinoma-appearing regions proved their common origin through shared *TERT* promoter mutation, *CDK4* amplification, and gain of chromosome 7 plus loss of chromosome 10.

## Case Report

**Figure 1.**
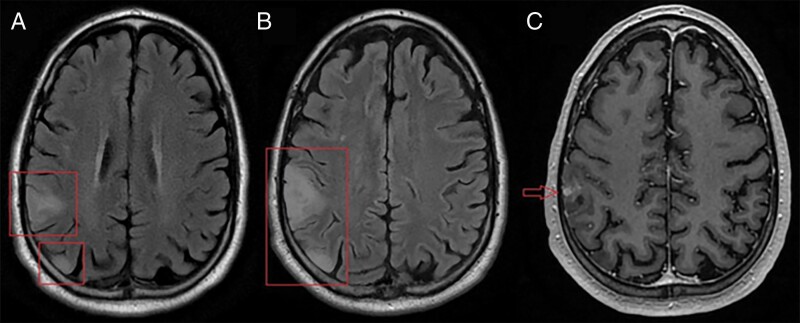
Axial magnetic resonance imaging. (A) T2 FLAIR imaging of 2 initial right parietal lobe masses. (B) T2 FLAIR imaging of the same masses, 3 months later. Both lesions infiltrate further into the parietal lobe and mild gyral swelling and effacement of adjacent sulci is seen. (C) Additionally, a nodular enhancing area is seen on the T1-weighted image.

The patient was a 64 year old man with a prior history of prostate adenocarcinoma and a non-convulsive seizure 3 years ago, with no further follow-up or additional seizures until another one caused a motor vehicle accident. Magnetic resonance imaging at an outside institution showed 2 right parietal lobe masses, one laterally and one posteriorly, both suspected to be low-grade glioma (Figure 1A). Three weeks later, the patient experienced a third seizure and was placed on levetiracetam. Imaging was stable. Since magnetic resonance imaging findings were unchanged and seizures were well controlled, the patient was kept on surveillance. Abdominal and spinal CTs, and chest X-rays, were negative for suspicious lesions (not shown).

Three months later, despite a lack of clinical symptoms, both lesions showed growth and infiltration into the right parietal lobe (Figure 1B). A new area of nodular enhancement was also seen in the lateral lesion (Figure 1C). The patient underwent gross total resection.

## Pathology

**Figure 2.**
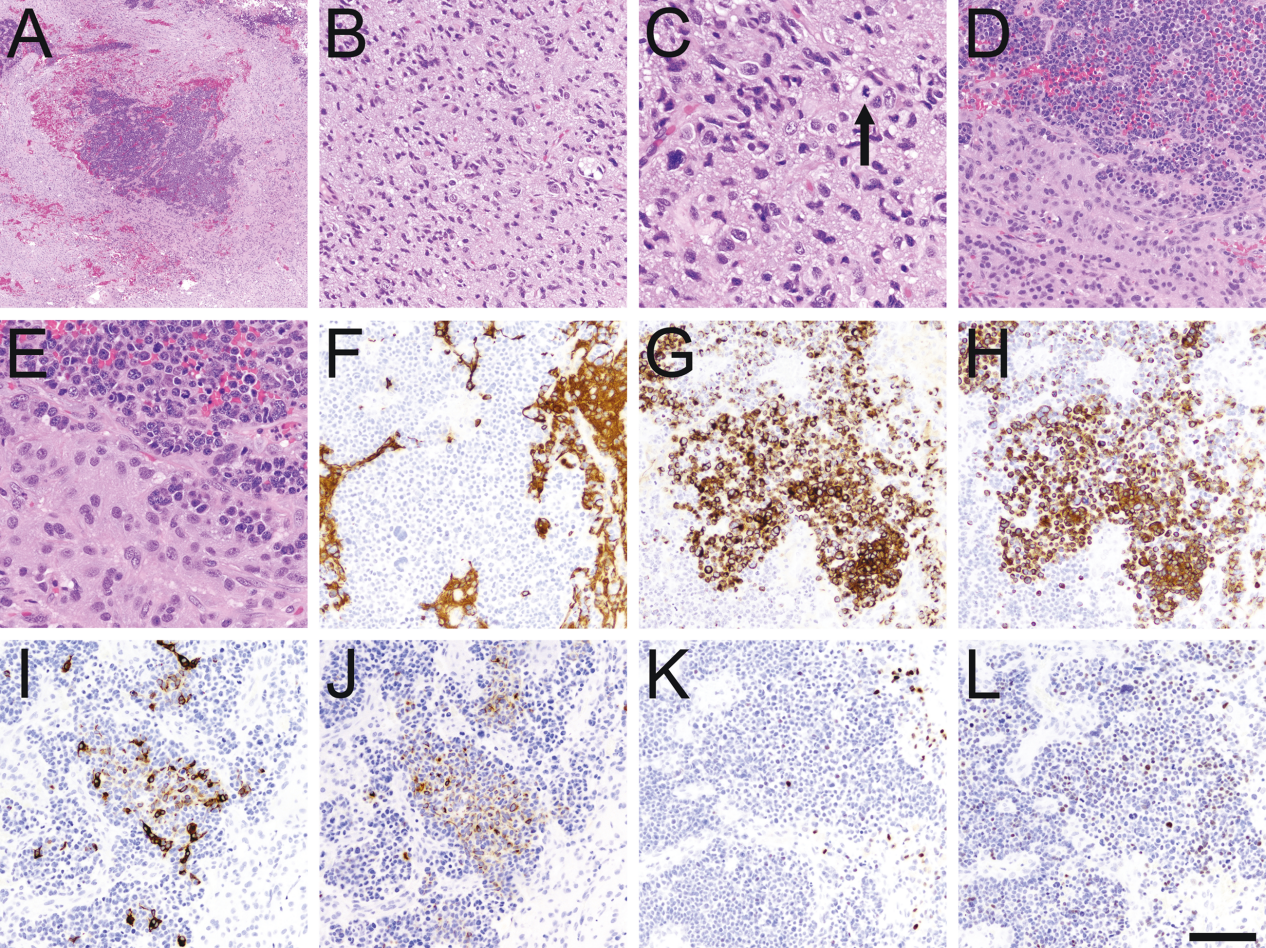
Histopathologic characteristics. (A) At low power, sharply demarcated islands of increased tumor cellularity were apparent. (B) Most of the tumor looked like a typical diffusely infiltrative high-grade glioma, including secondary structuring around neurons and mitoses (C, arrowhead). (D–E) Higher power views of the cellular islands showed cells with high nuclear: Cytoplasmic ratio, numerous mitoses, and abundant apoptosis. (F) The cellular islands were mostly negative for GFAP, although a few scattered cells were positive. The cellular islands were also variably positive for cytokeratin AE1/3 (G), CAM5.2 (H), cytokeratin 7 (I), and cytokeratin 20 (J). Some cells were positive for p63 (K) and TTF-1 (L). Scale bar in (L) = 500 microns in A, 50 microns in C and E, and 100 microns in B, D, and F-L.

Histopathology showed infiltrative high-grade glioma with scattered islands of epithelioid cells (Figure 2A–E). Immunohistochemical studies showed that those islands were largely negative for GFAP and positive for cytokeratins AE1/3, CAM2.5, CK7, CK20, p63, and TTF-1 clone 8G7G3-1 (Cellmark) (Figure 2F–L). Among other immunostains not shown, ATRX nuclear expression was retained in all tumor cells, and none of the tumor cells were positive for IDH1 R132H mutant protein, napsin, NKX3.1, CD45, synaptophysin, INSM1, SOX10 protein, p40, CDX2 protein, PSAP, or PSA. Nuclear accumulation of p53 was stronger in the epithelial regions. Ki-67 index was 50%–60% in areas of epithelial differentiation and 1%–2% in infiltrating glioma cells; the latter was low but within previously documented ranges for glioblastoma.^4^

## Molecular Diagnostics

Given the morphologic and immunohistochemical findings, glioma, and epithelial regions were separated from each other by microdissection and analyzed by next-generation sequencing, methylation profiling, fusion screening, and copy number array. Both the glioma and epithelial regions had a c.-124C > T *TERT* promoter mutation and *CDK4* amplification, as well as gain of chromosome 7 and loss of 10 (Table 1). The glioma region also had an *EGFR* mutation and amplification and *MDM2* amplification, while the epithelial region had a *TP53* mutation and *FGFR3::TACC3* fusion. Both regions lacked *MGMT* promoter methylation and *IDH1/2* mutations.

**Table 1.** Molecular Comparison Between Conventional and Epithelial Regions

Regions of high-grade glioma morphology (%: variant allelic fraction)	Regions of epithelial differentiation (%: variant allelic fraction)
*TERT* variant (c.-124C > T), 36%	*TERT* variant (c.-124C > T), 52%
*CDK4* amplification (21.0 copies)	*CDK4* amplification (25.0 copies)
Gain of chromosome 7	Gain of chromosome 7
Loss of chromosome 10	Loss of chromosome 10
*EGFR* variant (c.2320G > A), 24%	No *EGFR* variant
*EGFR* amplification (73.0 copies)	No *EGFR* amplification
*MDM2* amplification (35.0 copies)	No *MDM2* amplification
No *TP53* variant	*TP53* variant (c.724T > A), 61%
No fusion transcript	*FGFR3::TACC3* fusion
Methylation class “glioblastoma, IDH wild type” (0.99)	Methylation class “brain” (0.96), subclass “glioblastoma” (0.8)

By genomic methylation profiling on an Illumina EPIC V2 chip using our in-house classifier,^5^ the glioma region matched with methylation class “glioblastoma, IDH wild type” (score = 0.99) with subclass “RTK II” (score = 0.81). In contrast, the epithelial region did not match, with the closest being glioblastoma, IDH wild type (score = 0.68). However, when the same methylation data from the epithelial region was run with an in-house classifier for cancer of unknown primary, it matched as methylation class “brain” (score = 0.96) with subclass “glioblastoma” (score = 0.8).

## Discussion

Glioblastoma with bona fide epithelial differentiation is extremely rare and was first described in a 1988 case report.^6^ It is not to be confused with *epithelioid* glioblastoma, a recognized variant in the 2021 WHO classification of brain tumors. As in the presented case, glioblastoma with epithelial differentiation should have epithelial morphology and immunopositivity for epithelial markers, such as cytokeratins CAM2.5, AE1/3, 7, and 20.^2^ While cytokeratin AE1/3 antibodies can sometimes cross-react with GFAP, that did not occur in this case (compare Figure 2F with 2G).^7^ Such tumors also tend to have *TP53* mutations and some sort of cell cycle-activating alteration, most commonly *CDKN2A/B* deletion.^2^ In the current case, that activation was in the form of *CDK4* amplification.

Glioblastoma with epithelial differentiation can easily be mistaken for tumor-to-tumor metastasis, a rare event in which a tumor is invaded by a distant unrelated neoplasm. It is distinct from collision tumors where different neoplasms in the same tissue maintain definite borders. The most common intracranial neoplasms acting as recipients are meningiomas, but tumor-to-tumor metastasis is observed in glioblastoma as well.^8^ Carcinomas (ie, epithelial cell neoplasms) are by far the most common donors, and before molecular diagnostics were in widespread use, patients with glioblastoma with epithelial differentiation were occasionally misdiagnosed or received unnecessary testing for distant tumors.^3,9^

As demonstrated by this case, molecular diagnostics are extremely helpful in differentiating between glioblastoma with epithelial differentiation and tumor-to-tumor metastasis. Prior reports have proven clonality between the epithelial and conventional regions of glioblastomas based on shared *TP53* mutations, among other alterations.^10–12^ In the current case, the common driver alterations appeared to be the *TERT* promoter mutation and *CDK4* amplification. EGFR-driven subclones developed along a conventional glioblastoma morphology, while subclones with *TP53* and *FGFR3::TACC3* fusion became epithelial. The *FGFR3::TACC3* fusion has been described in a variety of gliomas, including glioblastomas, and can produce a variety of morphologies and outcomes.^13,14^ To the best of our knowledge, *FGFR3::TACC3* has not previously been associated with epithelial differentiation in gliomas, although it does occur in carcinomas.^15^

Initial methylation profiling of the epithelial region, which failed to match with glioblastoma, was done with a reference library that only contained primary CNS tumors,^5^ and was under-representative of glioblastomas with epithelial differentiation. However, when using a reference library that included glioblastoma as well as carcinomas, the classifier was able to correctly discern that the epithelial regions still retained more epigenomic similarity to glioblastomas than to non-CNS cancers. This underscores the importance of considering alternative methylation classifier libraries if a match is not found with the first library that is used.

In sum, this case demonstrates the ability of molecular testing to avoid misdiagnosing glioblastoma with epithelial differentiation as tumor-to-tumor metastasis. To the best of our knowledge, it is also the first time that the *FGFR3::TACC3* fusion has been described in such a tumor, specifically in the epithelial region. While FGFR3-targeting therapeutics exist, their utility in this case is unclear since the fusion only exists in the epithelial subset of the tumor.
